# Takotsubo Syndrome – Predictable from brain imaging data

**DOI:** 10.1038/s41598-017-05592-7

**Published:** 2017-07-14

**Authors:** Carina Klein, Thierry Hiestand, Jelena-Rima Ghadri, Christian Templin, Lutz Jäncke, Jürgen Hänggi

**Affiliations:** 1 Division Neuropsychology, Department of Psychology, University of Zurich, Zurich, Switzerland; 2 University Heart Center, Department of Cardiology, University Hospital Zurich, Zurich, Switzerland; 3 International Normal Aging and Plasticity Imaging Center (INAPIC), University of Zurich, Zurich, Switzerland; 4 Center for Integrative Human Physiology (ZIHP), University of Zurich, Zurich, Switzerland; 5 University Research Priority Program (URPP), Dynamic of Healthy Aging, University of Zurich, Zurich, Switzerland; 6 Department of Special Education, King Abdulaziz University, Jeddah, Saudi Arabia

## Abstract

Takotsubo syndrome (TTS) is characterized by acute left ventricular dysfunction, with a hospital-mortality rate similar to acute coronary syndrome (ACS). However, the aetiology of TTS is still unknown. In the present study, a multivariate pattern analysis using machine learning with multimodal magnetic resonance imaging (MRI) data of the human brain of TTS patients and age- and gender-matched healthy control subjects was performed. We found consistent structural and functional alterations in TTS patients compared to the control group. In particular, anatomical and neurophysiological measures from brain regions constituting the emotional-autonomic control system contributed to a prediction accuracy of more than 82%. Thus, our findings demonstrate homogeneous neuronal alterations in TTS patients and substantiate the importance of the concept of a brain-heart interaction in TTS.

## Introduction

*“Give sorrow words; the grief that does not speak*

*Whispers the o’er-fraught heart and bids it break”*

(*William Shakespeare, MacBeth, Act IV, Scene III, lines 245*–*246*)

More than a quarter of a century after its first description in 1990^1^, TTS is still a largely unknown disease^2^. Given that external stressors are a unique feature that provoke a TTS event, it is conceivable that TTS is triggered by neuronal alterations in the limbic system, presuming an emotional hypersensitivity or disturbed emotional processing in these patients. In fact, first studies provide insights into neuronal alterations in brain regions constituting the limbic and the central autonomic nervous system^3, 4^. Research in the field of neuroimaging and TTS is rather sparse and limited to studies applying hypotheses-driven mass-univariate statistics^3, 4^. However, with this statistical approach it is not possible to draw conclusions about whether patients with TTS show homogeneous neural alterations, i.e. alterations that are consistently observable across all patients. Thus, in contrast to this classical mass-univariate statistical analyses of neuroimaging data, here, we apply a multivariate pattern analysis in a machine learning framework. This approach offers the advantage of delineating regularities of anatomical and neurophysiological measures. These regularities are then used to discriminate between different conditions or, as here, subject groups. Thereby, it also provides information about the consistency of the statistical pattern across the single subjects^5^. Therefore, the aim of the present study was to identify predictors for the presence of TTS based on different modalities of MRI data.

## Results

**Figure 1:**
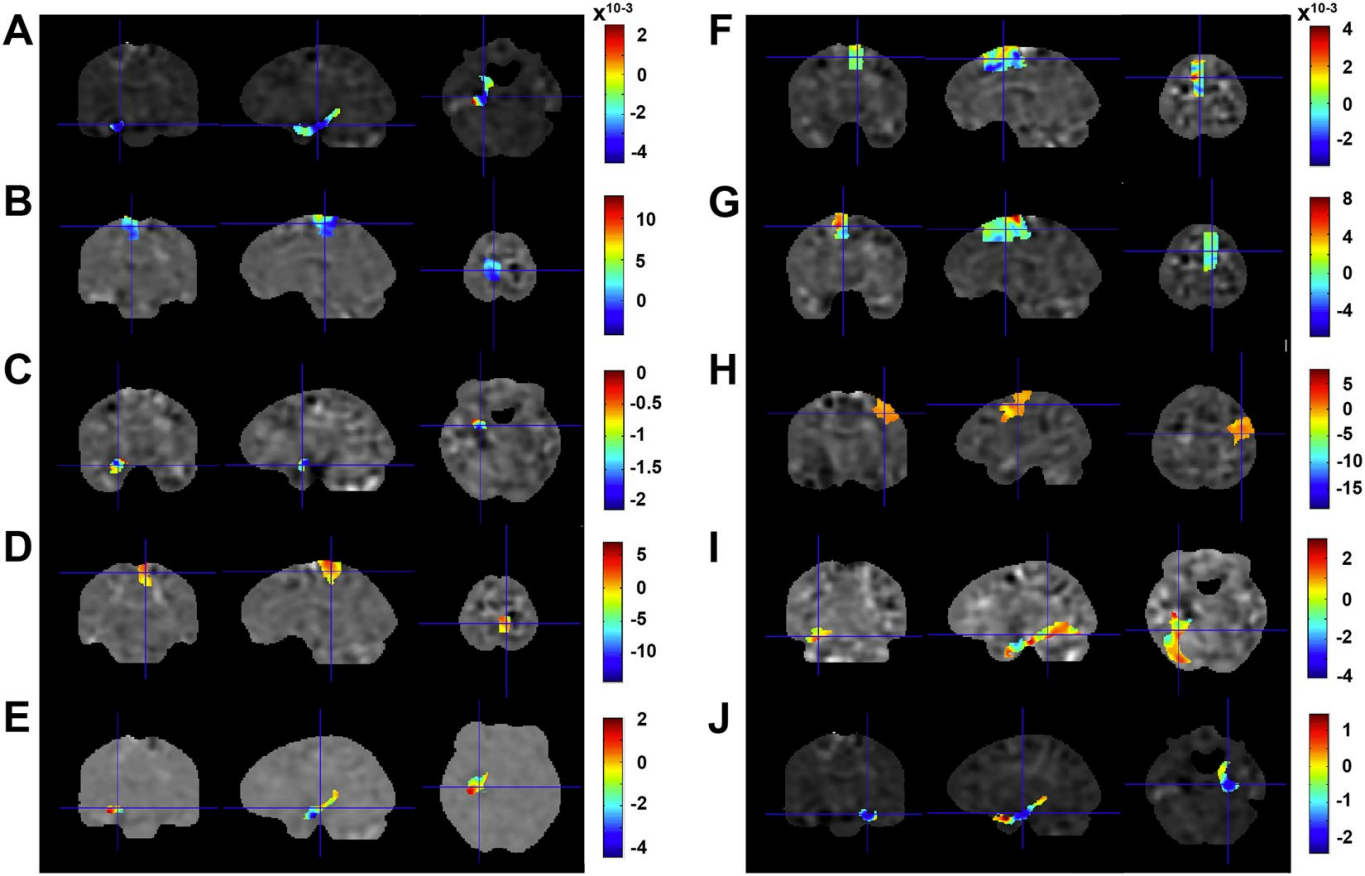
Model weights (colour bar) obtained with the support vector machine algorithm displayed for the brain regions with the highest weights for group classification based on FA (averaged across all cross-validation folds): Left parahippocampal gyrus (A), left paracentral lobe (B), left amygdala (C), right paracentral lobe (D), left hippocampus (E), left (F) and right (G) supplementary motor areal, right precentral gyrus (H), left fusiform gyrus (I), and right parahippocampal gyrus (J).

Regarding diffusion-based parameters, our results delivered an accuracy of 82% (p = 0.002) with a sensitivity of 93% (p = 0.001) and a specificity of 71% (p = 0.06) for fractional anisotropy (FA), a measure of white matter (WM) integrity. WM fibre density showed an accuracy of 75% (p = 0.026), a sensitivity of 79% (p = 0.023), and a specificity of 71% (p = 0.074), whereby both parahippocampal gyri, both amygdalae, both paracentral lobuli, left hippocampus, and right posterior cingulate cortex showed highest predictive power (for details, see Supplementary Table S3 and Fig. 1). Axial, radial, and mean diffusivity did not discriminate between groups. Analysis of resting state functional magnetic resonance imaging (rsfMRI) measures, such as the fractional amplitude of low frequency fluctuations (fALFF) showed an accuracy of 75% (p = 0.009), a sensitivity of 81% (p = 0.009), and a specificity of 69% (p = 0.061). Regional homogeneity (ReHo) and ALFF discriminated less accurately; both had accuracies of 72% (ReHo p = 0.015; ALFF p = 0.021), sensitivities of 75% (ReHo p = 0.015; ALFF p = 0.017), and specificities of 69% (ReHo p = 0.046; ALFF p = 0.017). In this context, the bilateral parahippocampal gyri, paracentral lobuli, superior parietal lobuli, and left insula showed the highest predictive values (for details, see Supplementary Table S4). Voxel-based morphometry (VBM)-derived local WM volume showed an accuracy of 63% (p = 0.088), a sensitivity of 74% (p = 0.023), and a specificity of 53% (p = 0.432) with the left precentral gyrus, both paracentral lobuli, bilateral supplementary motor areas, inferior temporal gyri, and superior parietal lobe as the strongest predictive regions. In contrast, VBM-derived local gray matter (GM) volume did not predict group membership (for details, see Supplementary Table S5). Figure 1 shows the brain regions with the highest weights derived from FA, the best predictive brain measure for group classifications (based on accuracy).

**Figure 2:**
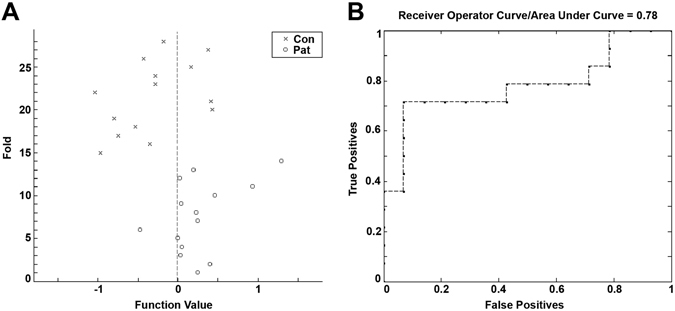
Prediction values (**A**) and the Receiver Operating Characteristic (ROC) curve (**B**) of group classification based on FA (averaged across all folds).

The respective distribution and the receiver operating characteristic (ROC) curve of the group classification is depicted in Fig. 2 (for details regarding all MRI modalities see Supplementary Table S2). Multi-kernel learning analyses across all measures from one MRI-modality and an analysis across all measures from all modalities (diffusion tensor imaging (DTI), VBM, and rsfMRI) revealed comparable sensitivity, specificity and accuracy values, being however below the best value of the single measures (DTI-all: accuracy: 79%, p = 0.01; sensitivity: 79%, p = 0.14; specificity: 79%, p = 0.26; VBM-all: accuracy: 58%, p = 0.15; sensitivity: 58%, p = 0.55; specificity: 58%, p = 0.48; rsfMRI all: accuracy: 78%, p = 0.01; sensitivity: 81%, p = 0.24; specificity: 75%, p = 0.27; all modalities: accuracy: 71%, p = 0.04; sensitivity: 67%, p = 0.32; specificity: 75%, p = 0.36).

## Discussion

We show for the first time that TTS patients demonstrate specific homogeneous anatomical and neurophysiological features in brain regions mainly responsible for controlling heart functions^6^ and emotional processing by applying a multivariate pattern analysis approach based on machine learning^7, 8^. The identified brain regions are also core domains of a network controlling emotions, cognitive, and sensorimotor functions. Most importantly, several of these areas form the “central part” of the autonomic nervous system controlling cardiovascular functions via the sympathetic and parasympathetic nervous systems^7^. Brain regions which are most strongly involved in the modulation of the sympathetic nervous system were the supplementary motor area, left paracentral gyrus, left superior parietal lobe, putamen and hippocampus, while the right precentral gyrus, precuneus, and medial temporal gyrus drive activations of the parasympathetic system. Brain areas regulating both divisions were the left amygdala, angular gyrus, and left insula^7^.

Recently, Templin and colleagues have demonstrated that the prevalence of psychiatric comorbidities such as depression, fear, anxiety, and stress is substantially increased in TTS patients^2^. In fact, patients afflicted with depression and/or anxiety demonstrate functional and structural adaptations in most of the brain regions, which we found to be predictive for the presence of TTS. In this context, a core area of the identified TTS-specific network, the amygdala, is known to be strongly involved in cardiac control and also plays a pivotal role in fear conditioning^9^, mental^3^ and posttraumatic stress^10^, depression^11^ as well as anxiety disorders^12^. However, since the examined TTS patients in the present study did not show differences in anxiety, depression, or stress symptoms compared to the controls, our results do not seem to be driven by the presence of possible psychiatric disorders. The insula, which, among others, plays a role in the control of the spontaneous baroreflex is a further core region that we identified to be specific for TTS^6^. Strokes or seizures in the insula or amygdala can lead to severe cardiac arrhythmias and other autonomic dysfunctional manifestations^13^.

Although we found specific anatomical and neurophysiological features in the above-mentioned brain areas in TTS patients, it is currently not clear how these features are related to cardiac dysfunction. Based on the literature, several direct and/or indirect effects from these brain regions on the cardiac system can be suggested. Patients suffering from depressive disorders show an impaired neuronal norepinephrine reuptake from the synaptic cleft^14^. This in turn might lead to prolonged sympathetic neural signalling and subsequently to an excessive catecholamine release resulting in myocardial stunning and contractile dysfunction^15^. Based on these and further findings, it has been proposed that an impaired control of the spontaneous sympathetic baroreflex (mediated via the insula) might contribute to the pathogenesis of TTS^16^. Since we also identified various brain regions involved in the parasympathetic control of heart functioning to be predictive for the presence of TTS, we speculate that an impaired mutual interference of the parasympathetic and sympathetic nervous system could also be an underlying cause of TTS. Thus, the consequence could be a parasympathetic discharge alongside a sole impairment of the baroreflex control of sympathetic activity.

Besides the insula and the amygdala, we also identified orbitofrontal areas substantially contributing to our classification results. Previously, it has been shown that these areas have an impact on cardiovascular functions via a tonic inhibition of the amygdala, thereby down-regulating the sympatho-excitatory neurons in the medulla^17, 18^. As summarized by Beissner and colleagues^7^, the brain regions involved in the control of vegetative functions also play a crucial role in executive and salience processing, and are to a certain extent part of the default-mode network. Interestingly, in this context, a currently published rsfMRI study showed increased connectivity of areas in the precuneus and decreased connectivity in the ventromedial prefrontal cortex of TTS patients compared to healthy controls, indicating an increased involvement of the default mode network in TTS^4^. Given that the TTS patients of that study reported enhanced anxiety levels and often experienced negative affects, Sabiz and colleagues suggested that this increased focus on internal and self-regulation processes might reflect an inefficient emotion regulation mechanism in the patients^4^.

As stated above, one of the parasympathetic brain regions predictive for TTS is the angular gyrus. The angular gyrus is often described as a convergence zone integrating and binding information into a multimodal system^19^. Structurally it is connected to other brain areas that we found being predictive for TTS, such as the middle and inferior temporal regions via the middle longitudinal^20, 21^ and the arcuate fasciculus^22^. It is also connected with the precuneus and the superior frontal gyrus via the occipito-frontal fasciculus^23^ and with the hippocampus^24^ and parahippocampal gyrus^25^ via the inferior longitudinal fascicle. On the functional level, the angular gyrus has been reported to be involved in the default-mode network^26–28^, theory-of-mind^27, 29^, in task-free semantic and conceptual processes^30^ as well as in conflict resolution^31^. Due to the diversity of brain functions in which the angular gyrus is involved in, it is difficult to define its underlying role in TTS. However, the control of parasympathetic activity, information integration and (emotional) conflict resolution seems convincing.

Besides an increased prevalence for anxiodepressive disorders and stress, Templin and colleagues have also shown that rates of neurologic or psychiatric disorders were higher in a substantial number of TTS patients (55.8%) compared to ACS patients (25.7%)^2^. This raises the question whether brain alterations involved in the pathophysiology of TTS are only present during the acute phase, or perhaps pre- or post-existent. In this context, literature suggests that a history of neurological and psychiatric disorders might induce functional and structural changes within the brain leading to sympathetic hyperactivation, excessive catecholamine release and subsequently TTS^3, 4, 16^. Therefore, pre-existing alterations of the respective brain areas might serve as a potential risk factor for TTS.

## Conclusion and Limitations

Our results reveal that brain regions that are primarily involved in cardiac control and emotional processing are homogeneously altered across TTS patients compared to healthy age- and gender-matched controls. This finding underscores an important role of the brain-heart axis in TTS. Furthermore, given the fact that we identified brain regions to be predictive for TTS that are involved in the control of both the parasympathetic and the sympathetic nervous system, this may suggest a parasympathetic discharge or an impaired interference of these two systems in TTS, rather than only a sympathetic hyperactivity. In addition, our findings lead us to conclude that functional as well as structural MRI measures are promising candidates for the classification of TTS patients. Based on the ROC-AUC (area under curve) values, DTI (all, FA, and density) and rsfMRI are the most promising MRI measures for identifying TTS patients and distinguishing them from healthy controls. We acknowledge constraints about the generalizability of our findings since the TTS patient cohort is relatively small. However, given a TTS prevalence of 2–3% of patients with suspected acute coronary syndromes^32, 33^ the number of TTS patients in the present study is nevertheless moderately large enough for a single centre neuroimaging study using MRI. To provide a deeper understanding about the exact role of the limbic system and the autonomous nervous system in the development of TTS, further prospective studies are required investigating for example acute TTS patients or applying a longitudinal study design.

## Materials and Methods

### Subjects

Twenty TTS female patients (diagnosed after the Mayo Clinic criteria; mean age = 65.32 years, standard deviation, SD = 14.26 years) selected from the International Takotsubo Registry (www.takotsubo-registry.com) of the leading hospital and 19 healthy control women (derived from the l-HAB (longitudinal healthy aging brain) database^34^ of the International Normal Aging and Plasticity Imaging Center (INAPIC) of the University of Zurich (mean age = 67.42 years, SD = 14.15 years)) matched by sex, age (t_(36)_ = −0.46, p = 0.65), handedness^35^ and the score of the mini mental state (MMSE; t_(36)_ = −1.12, p = 0.27)^36^ examination, participated in the study. The majority of the participants of the l-HAB database were recruited at the “Seniorenuniversität”, a university program for interested and motivated elderly (www.seniorenuni.uzh.ch). The median of the time between the TTS event and the acquisition of the MRI scans was 168 days (SD = 266.74 days; minimum = 56 days, maximum = 885 days in between; 25th percentile = 116 days, 75th percentile = 536 days). Levels of anxiety and depression were evaluated using the hospital anxiety and depression scale (HADS; anxiety t_(33)_ = 0.78, p = 0.44; depression t_(33)_ = −0.43, p = 0.67)^37^. For detailed information on behavioural, demographic and clinical parameters, see Table 1. None of the participants reported any history of head injuries, neurosurgery, current drug or alcohol abuse or contraindications to MRI. Nineteen patients and 19 control subjects entered data analysis of the T1-weighted images (one patient was excluded because of incidental brain anomalies) and 14 patients and 14 healthy controls entered data analysis of the diffusion-weighted images (five patients were unable to finish the diffusion-weighted sequence or the images contained excessive MRI-related motion artefacts). Resting state functional MRI data were analysed for 16 TTS patients and 16 healthy controls (three patients were unable to finish the resting state functional MRI sequence). Written informed consent was obtained from all participants prior to the study enrolment. The present study was approved by and all applied methods are in accordance with the local ethics committee (“Kantonale Ethikkommission Zürich”; www.kek.zh.ch) and has been conducted accordingly to the principles expressed in the declaration of Helsinki.

**Table 1:** Demographic, clinical and behavioural characteristics.

Characteristic	TTS (n = 19)	Control (n = 19)	p-value
Age (years, n = 38)	65.32 ± 14.26	67.42 ± 14.15	0.65
Female sex no. (%)	19 (100)	19 (100)	
Handedness (frequency: right\/left)	17/2	17/2	
Time between TTS and MRI (days, median)	168 ± 266.74		
MMSE (max. 30, n = 38)	28.74 ± 2.08	29.32 ± 0.89	0.27
HADS anxiety (max. 21, n = 35)	5.29 ± 3.92	4.39 ± 2.93	0.44
HADS depression (max. 21, n = 35)	3.47 ± 4.05	3.00 ± 2.22	0.67
Chestpain no. (%)	13 (68.4)		
Dyspnea no. (%)	11 (57.9)		
High-sensitivity troponin T (ng/mL, n = 14)	0.40 ± 0.22		
Creatine kinase (IU/L)	240.8 ± 199.9		
NT-proBNP (pg/mL, n = 15)	8,113.0 ± 9,739.0		
ST-segment changes (no. %, n = 18)	8 (44.4)		
Heart rate (beats/min, n = 17)	74.6 ± 13.9		
Systolic blood pressure (mmHg, n = 17)	123.2 ± 25.3		
Left ventricular ejection fraction (%)*	44.9 ± 13.8		
Left ventricular end diastolic pressure (mmHg, n = 14))	23.7 ± 4.1		
Coronary artery disease (no. %, n = 17)	4 (23.5)		
Recurrence of TTS no. (%)	4 (21.1)		

Characteristics were compared between groups using two-sample t-Tests. Plus-minus values are means ± standard deviation. Abbreviations: HADS, Hospital anxiety and depression scale; MMSE, mini mental state examination; max., maximum; n, number of subjects.

*Data regarding the left ventricular ejection fraction were obtained either during catheterization or echocardiography. If both results were available, data obtained during catheterization are reported.

### Magnetic resonance imaging data acquisition

The technical details of the magnetic resonance imaging sequences can be found in the Supplementary Methods.

### Data preprocessing for voxel-based morphometry

We used VBM^38, 39^ to investigate local GM, WM, and cerebrospinal fluid (CSF) volume. The computational anatomy toolbox (CAT12, release 825, http://dbm.neuro.uni-jena.de/cat12/) was applied using the statistical parametric mapping (SPM12, release 6470, http://www.fil.ion.ucl.ac.uk/spm/) software running in MATLAB (release 2013b; http://www.mathworks.com/). CAT12 is the new name for the further-developed VBM12 toolbox. Default parameters were used except for the voxel resolution that was set to 1 × 1 × 1 mm^3^. VBM preprocessing includes bias field correction, tissue class segmentation, spatial normalisation, Jacobian determinant modulation, and smoothing with a Gaussian kernel of full-width at half maximum (FWHM) of 8 mm. These maps were then subjected to multivariate statistical analyses (see below).

### Preprocessing of diffusion tensor imaging data

Preprocessing of the diffusion-weighted MRI data was performed with FSL tools (FMRIB software library; version 5.0.6; http://www.fmrib.ox.ac.uk/fsl/)^40^ such as the FDT (FMRIB diffusion toolbox; version 3.0)^41^ and tract-based spatial statistics (TBSS)^42^. For deterministic fibre tractography we used the Diffusion Toolkit (DTK, version 0.6.2.1) and TrackVis software (version 0.5.2.1; http://trackvis.org/)^43^.

Head motion parameters of the DTI data have been extracted and compared between groups (see Supplementary Methods).

To construct fractional anisotropy, mean, axial, and radial diffusivity maps as well as white matter fibre density maps, the following fully automated preprocessing steps were realized: 1) In a first step, a binary brain mask was created using FSL’s brain extraction tool (BET). This mask was used in later steps to exclude non-brain tissue. 2) Eddy current distortions and head movements were corrected using the EDDY_CORRECT tool of FDT. 3) Diffusion gradients were adjusted for rotations introduced by the eddy current and head movement corrections. 4) The preprocessed DTI data were then subjected to TBSS as well as the DTK to compute voxel-wise diffusion tensors and to construct the (principal) eigenvector and eigenvalue maps as well as a map of fractional anisotropy, mean, axial, and radial diffusivity, respectively. 5) These diffusivity maps were then spatially normalized using TBSS routines and the non-skeletonized version of these maps were smoothed with a FWHM Gaussian kernel of 8 mm and subjected to multivariate statistical analyses (see below). The further steps applied to the DTI data to obtain fiber density maps are described in the Supplementary Methods.

### Preprocessing of resting state functional MRI data

Functional MRI data were preprocessed according to standard procedures with the DPARSFA toolbox (version 3.1, as implemented in DPABI toolbox version 1.2, rfmri.org/dpabi) using the functions of statistical parametric mapping software (SPM8, fil.ion.ucl.ac.uk/spm/software/spm8/). Preprocessing consisted of the following steps: 1) Coregistration of the T1-weighted image onto the functional images, 2) slice timing correction, 3) realignment combined with the extraction of the frame-wise displacement parameters according work by Power and colleagues^44^ for later comparison of head motion parameters between groups, 4) estimation of linear and non-linear spatial transformations of the T1-weighted MRI image using the unified segmentation approach as implemented in SPM8, 5) application of estimated transformations onto the functional images, 6) voxel re-sampling to 2 × 2 × 2 mm^3^, 7) smoothing with a Gaussian kernel of 4 mm full width at half maximum, 8) filtering to reduce physiological noise (frequencies 0.01 < *f* < 0.1 Hz passed the filter) and 9) regressing out the variance of nine nuisance covariates, i.e. the six parameters from head motion correction (three translation and three rotation parameters) as well as the global mean signal, white matter signal, and cerebrospinal fluid signal. Although there is an ongoing dispute about whether regressing out the global mean signal in rsfMRI data analyses is beneficial or affects data detrimentally^45^, we applied global mean signal regression because it has also be shown that the global mean signal regression is very effective in controlling movement-related artefacts in functional connectivity measures^44, 46^.

Specific parameters for regional homogeneity (ReHo) were extracted before smoothing^47^. ReHo is a measure of functional connectivity in a voxel-by-voxel manner through the calculation of Kendall’s *W* (or coefficient of concordance (KCC)) assessing temporal functional connectivity correlations in a given voxel with those of its nearest neighbours^48^. Larger ReHo values indicate higher regional synchronization and can inform about functional homogeneity within brain regions with heterogeneous functional properties.

In contrast to ReHo, amplitude of low frequency fluctuations (ALFF; integrating the square root of a power spectrum in the low frequency range) and fractional ALFF (fALFF; the ratio of the low-frequent power spectrum between 0.01 and 0.08 Hz to that of the entire frequency range between 0 and 0.25 Hz)^49^ investigates local brain signal variability in the frequency domain by calculating low frequency power of the single voxels^50^.

The Fisher’s z-transformed ReHo, ALFF, and fALFF maps were smoothed by additionally 4 mm FWHM (resulting in total 8 mm smoothing) and then subjected to multivariate statistical analyses (see below).

### Statistical analyses

Group differences regarding clinical and demographic characteristics (see Table 1), global brain measures (Supplementary Table S1) were evaluated using student’s t-tests for independent samples and conducted with the IBM SPSS Statistics software (version 22, http://www-01.ibm.com/software/analytics/spss/products/statistics/).

For multivariate statistics, a support vector machine adopted from the field of machine learning algorithms has been applied. We used the Pattern Recognition for Neuroimaging data Toolbox (PRoNTo, http://www.mlnl.cs.ucl.ac.uk/pronto/)^5^ for classifying groups.

Within PRoNTo, we used the standard mask provided by the tool as a first-level mask for all classifications. For constructing the feature sets, no additional mask was applied. We used hyperparameter optimisation in the range between 0.001 and 1,000 in steps of 10 and a leave-one-subject-out (LOSO) cross-validation scheme was applied. For deriving p-values of the accuracy, sensitivity, and specificity, 1,000 nonparametric permutations of the group label were performed for the single modalities measures. Due to computational efficiency reasons, permutations of the group label were performed using only 100 permutations each for model estimation including all single parameters per modality as well as for model estimation across all measures of all modalities (i.e. all DTI, rsfMRI and VBM measures included in one model; see Supplementary Table S2).

### Data availability

The datasets generated during and/or analysed during the current study are available from the corresponding author on reasonable request.

## Electronic supplementary material

Supplementary Information

## References

[1] Sato, H. T. H., Uchida, T., Dote, K. & Ishihara, M. Tako-tsubo-like left ventricular dysfunction due to multivessel coronary spasm. In Kodama, K., Haze, K., Hori, M. eds. *Clinical aspect of myocardial injury*: *from ischemia to heart failure. Tokyo: Kagakuhyoronsha Publishing* (*in Japanese*), 56–64 (1990).

[2] Templin C (2015). Clinical Features and Outcomes of Takotsubo (Stress) Cardiomyopathy. N Engl J Med.

[3] Pereira VH (2016). Central autonomic nervous system response to autonomic challenges is altered in patients with a previous episode of Takotsubo cardiomyopathy. Eur Heart J Acute Cardiovasc Care.

[4] Sabisz A (2016). Brain resting state functional magnetic resonance imaging in patients with takotsubo cardiomyopathy an inseparable pair of brain and heart. Int J Cardiol.

[5] Schrouff J (2013). PRoNTo: pattern recognition for neuroimaging toolbox. Neuroinformatics.

[6] Palma JA, Benarroch EE (2014). Neural control of the heart: recent concepts and clinical correlations. Neurology.

[7] Beissner F, Meissner K, Bar KJ, Napadow V (2013). The autonomic brain: an activation likelihood estimation meta-analysis for central processing of autonomic function. J Neurosci.

[8] Craig AD (2005). Forebrain emotional asymmetry: a neuroanatomical basis?. Trends Cogn Sci.

[9] Hartley CA, Fischl B, Phelps EA (2011). Brain structure correlates of individual differences in the acquisition and inhibition of conditioned fear. Cereb Cortex.

[10] Ganzel BL, Kim P, Glover GH, Temple E (2008). Resilience after 9/11: multimodal neuroimaging evidence for stress-related change in the healthy adult brain. Neuroimage.

[11] Greening SG, Osuch EA, Williamson PC, Mitchell DG (2014). The neural correlates of regulating positive and negative emotions in medication-free major depression. Soc Cogn Affect Neurosci.

[12] Milham MP (2005). Selective reduction in amygdala volume in pediatric anxiety disorders: a voxel-based morphometry investigation. Biol Psychiatry.

[13] Benarroch EE (1993). The central autonomic network: functional organization, dysfunction, and perspectives. Mayo Clin Proc.

[14] Barton DA (2007). Sympathetic activity in major depressive disorder: identifying those at increased cardiac risk?. J Hypertens.

[15] Lyon AR, Rees PS, Prasad S, Poole-Wilson PA, Harding SE (2008). Stress (Takotsubo) cardiomyopathy–a novel pathophysiological hypothesis to explain catecholamine-induced acute myocardial stunning. Nat Clin Pract Cardiovasc Med.

[16] Vaccaro A (2014). Direct evidences for sympathetic hyperactivity and baroreflex impairment in Tako Tsubo cardiopathy. PLoS One.

[17] Thayer JF, Lane RD (2009). Claude Bernard and the heart-brain connection: further elaboration of a model of neurovisceral integration. Neurosci Biobehav Rev.

[18] Wager TD (2009). Brain mediators of cardiovascular responses to social threat: part I: Reciprocal dorsal and ventral sub-regions of the medial prefrontal cortex and heart-rate reactivity. Neuroimage.

[19] Damasio AR (1989). Time-locked multiregional retroactivation: a systems-level proposal for the neural substrate of recall and cognition. Cognition.

[20] Frey S, Campbell JS, Pike GB, Petrides M (2008). Dissociating the human language pathways with high angular resolution diffusion fiber tractography. J Neurosci.

[21] Makris N (2009). Delineation of the middle longitudinal fascicle in humans: a quantitative, *in vivo*, DT-MRI study. Cereb Cortex.

[22] Catani M, Jones DK, ffytche DH (2005). Perisylvian language networks of the human brain. Ann Neurol.

[23] Makris N (2007). The occipitofrontal fascicle in humans: a quantitative, *in vivo*, DT-MRI study. Neuroimage.

[24] Uddin LQ (2010). Dissociable connectivity within human angular gyrus and intraparietal sulcus: evidence from functional and structural connectivity. Cereb Cortex.

[25] Rushworth MF, Behrens TE, Johansen-Berg H (2006). Connection patterns distinguish 3 regions of human parietal cortex. Cereb Cortex.

[26] Laird AR (2009). Investigating the functional heterogeneity of the default mode network using coordinate-based meta-analytic modeling. J Neurosci.

[27] Spreng RN, Mar RA, Kim ASN (2008). The common neural basis of autobiographical memory, prospection, navigation, theory of mind, and the default mode: a quantitative meta-analysis. J Cogn Neurosci.

[28] Mazoyer B (2001). Cortical networks for working memory and executive functions sustain the conscious resting state in man. Brain Res Bull.

[29] Mar RA (2011). The neural bases of social cognition and story comprehension. Annu Rev Psychol.

[30] Shehzad Z (2009). The resting brain: unconstrained yet reliable. Cereb Cortex.

[31] Fan J, Flombaum JI, McCandliss BD, Thomas KM, Posner MI (2003). Cognitive and Brain Consequences of Conflict. Neuroimage.

[32] Templin C, Napp LC, Ghadri JR (2016). Takotsubo Syndrome: Underdiagnosed, Underestimated, but Understood?. J Am Coll Cardiol.

[33] Redfors B (2015). Mortality in takotsubo syndrome is similar to mortality in myocardial infarction - A report from the SWEDEHEART registry. Int J Cardiol.

[34] Zollig J (2011). Plasticity and imaging research in healthy aging: core ideas and profile of the International Normal Aging and Plasticity Imaging Center (INAPIC). Gerontology.

[35] Annett M (1970). A Classification of Hand Preference by Association Analysis. Br J Psychol.

[36] Folstein MF, Folstein SE, McHugh PR (1975). “Mini-mental state”. A practical method for grading the cognitive state of patients for the clinician. J Psychiatr Res.

[37] Zigmond AS, Snaith RP (1983). The hospital anxiety and depression scale. Acta Psychiatr Scand.

[38] Good CD (2001). A Voxel-Based Morphometric Study of Ageing in 465 Normal Adult Human Brains. Neuroimage.

[39] Ashburner J, Friston KJ (2000). Voxel-Based Morphometry–The Methods. Neuroimage.

[40] Smith SM (2004). Advances in functional and structural MR image analysis and implementation as FSL. Neuroimage.

[41] Behrens TEJ (2003). Characterization and propagation of uncertainty in diffusion-weighted MR imaging. Magn Reson Med.

[42] Smith SM (2006). Tract-based spatial statistics: voxelwise analysis of multi-subject diffusion data. Neuroimage.

[43] Park JH (2009). Detection of traumatic cerebral microbleeds by susceptibility-weighted image of MRI. J Korean Neurosurg Soc.

[44] Power JD, Barnes KA, Snyder AZ, Schlaggar BL, Petersen SE (2012). Spurious but systematic correlations in functional connectivity MRI networks arise from subject motion. Neuroimage.

[45] Wong CW, Olafsson V, Tal O, Liu TT (2012). Anti-correlated networks, global signal regression, and the effects of caffeine in resting-state functional MRI. Neuroimage.

[46] Yan CG (2013). A comprehensive assessment of regional variation in the impact of head micromovements on functional connectomics. Neuroimage.

[47] Zang Y, Jiang T, Lu Y, He Y, Tian L (2004). Regional homogeneity approach to fMRI data analysis. Neuroimage.

[48] Kendall, M. & Dickinson Gibbons, J. Rank Correlation Methods. *Oxford University Press; 5th edition*, doi:ISBN-13: 978-0195208375 (1990).

[49] Zou QH (2008). An improved approach to detection of amplitude of low-frequency fluctuation (ALFF) for resting-state fMRI: fractional ALFF. J Neurosci Methods.

[50] Yuan R (2013). Regional homogeneity of resting-state fMRI contributes to both neurovascular and task activation variations. Magn Reson Imaging.

