# Supplementary material for: Takotsubo Syndrome – Predictable from brain imaging data
Source: Sci Rep. 2017 Jul 14;7:5434. doi: 10.1038/s41598-017-05592-7 (PMC5511237; doi:10.1038/s41598-017-05592-7)
Supplement: Supplementary file 1 — Supplementary Information [file 41598_2017_5592_MOESM1_ESM.pdf]

Supplementary Information

Takotsubo Syndrome – Predictable from brain imaging data

Carina Klein[1*], Thierry Hiestand[2], Jelena-Rima Ghadri[2], Christian Templin[2], Lutz Jäncke[1,3-6¶], Jürgen Hänggi[1¶*]

[1] Division Neuropsychology, Department of Psychology, University of Zurich, Zurich, Switzerland

[2] University Heart Center, Department of Cardiology, University Hospital Zurich, Zurich, Switzerland

[3] International Normal Aging and Plasticity Imaging Center (INAPIC), University of Zurich, Zurich, Switzerland

[4] Center for Integrative Human Physiology (ZIHP), University of Zurich, Zurich, Switzerland

[5] University Research Priority Program (URPP), Dynamic of Healthy Aging, University of Zurich, Zurich, Switzerland

[6] Department of Special Education, King Abdulaziz University, Jeddah, Saudi Arabia

[¶] jointly supervised this work; * Corresponding authors

Supplementary Methods

Magnetic resonance imaging data acquisition

Magnetic resonance imaging (MRI) data acquisition took place between July 2013 and July 2014 on a 3.0 Tesla Philips Ingenia whole body scanner (Philips Medical Systems, Best, The Netherlands) equipped with a transmit-receive body coil and a commercial 15-elements transmit-receive head coil array that is capable of sensitivity encoding (SENSE). Data acquisition was done on the same scanner as used for data collection of the l-HAB database (MRI data of the control participants) and with the same MRI sequences.

A volumetric 3D T1-weighted gradient echo sequence (turbo field echo) image was measured with a spatial resolution of 1.0 x 1.0 x 1.0 mm^3 (acquisition matrix 240 x 240 pixels, 160 sagittal slices) and reconstructed to a spatial resolution of 0.94 x 0.94 x 1.0 mm^3 (reconstruction matrix 256 x 256 pixels, 160 sagittal slices). Further imaging parameters were: field of view (FOV) = 240 x 240 mm^2, echo time (TE) = 3.79 ms, repetition time (TR) = 8.38 ms, flip-angle α = 8°, and SENSE factor R = 1.5. Total acquisition time was 7 minutes and 29 seconds.

A diffusion-weighted spin echo echo-planar imaging (EPI) sequence was used to obtain diffusion-weighted scans with a measured and reconstructed spatial resolution of 2.0 x 2.0 x 2.0 mm^3 (acquisition and reconstruction matrix 112 x 112 pixels, 75 slices). Further imaging parameters were: FOV = 224 x 224 mm^2, TE = 55.0 ms; repetition time (TR) = 23.938 s, α = 90°; SENSE factor R = 2.0, b-value b = 1,000 s/mm^2, and number of averages = 1. Diffusion was measured along 32 non-collinear directions preceded by a non-diffusion-weighted volume (reference volume with b = 0 s/mm^2). Total acquisition time was 15 minutes and 6 seconds.

Beside these two structural MRI sequences, resting state functional MRI was also applied. A T2*-weighted gradient echo EPI sequence with a spatial resolution of 3.5 x 3.5 x 3.5 mm^3 (reconstructed 1.72 x 1.72 x 3.5 mm^3) was used to acquire images of resting-state functional MRI. Imaging parameters were: TR = 2 s; TE = 21 ms; FOV = 220 x 220 x 151 mm^3; flip-angle α = 76°; slice thickness = 3.5 mm; number of slices = 43; SENSE factor R = 2.0. Total acquisition time lasted 7 minutes and 40 seconds. During acquisition of resting-state data, participants were instructed to lie still with their eyes open and not to think of anything specific or fall asleep for 8 minutes.

MRI scans such as T2-weighted, proton-density-weighted, and fluid attenuated inversion recovery-weighted were additionally acquired in order to exclude brain anomalies. All T1-weighted and conventional MRI scans were evaluated by an imaging specialist (JH) and in cases of suspected brain anomalies or obvious imaging artefacts the subjects were excluded from the sample and addressed to an experienced neuroradiologist for further analysis in case of brain anomalies.

Preprocessing of diffusion tensor imaging data

Head motion is an issue in diffusion tensor imaging (DTI) and it has been shown that differential head motion between groups during DTI data acquisition could translate into artificial differences in connectivity measures. [1] Therefore, we extracted the relevant motion parameters provided by the TRACULA (TRActs Constrained by UnderLying Anatomy; https://surfer.nmr.mgh.harvard.edu/fswiki/Tracula) tool [2] and compared these parameters between groups. Neither the average volume-by-volume translations (mean/SD: 0.822/0.241 mm versus 0.748/0.148 mm for TTS and HC, respectively, p = 0.34 independent sample t-test) nor the average volume-by-volume rotations (mean/SD: 0.007/0.002 ° versus 0.006/0.002 ° for TTS and HC, respectively, p = 0.15) did significantly differ between groups. The parameters percent bad slices and the average dropout score did also not differ between groups (p = 0.14 and p = 0.27, respectively).

The first 5 steps of the DTI preprocessing are described in the main manuscript and the steps 6-12 applied to obtain fiber density maps are described here: 6) Deterministic tractography was conducted in TrackVis using the "brute force" approach with an interpolated streamline tracking algorithm. Twenty streamlines per voxel were propagated (using the -rseed option in DTK with value 20) and fibre tracking was stopped if FA was lower than 0.10 or if the turning angle of a streamline between two consecutive voxels was larger than 45°. This resulted in a whole-brain connectome comprised by about 2-3 million of streamlines depending on brain size including subcortical pathways and connections to the cerebellum. 7) The individual FA map was registered onto the FMRIB58-FA template, which is in correspondence with the MNI152 standard space, using FSL's linear image registration tool (FLIRT; http://fsl.fmrib.ox.ac.uk/fsl/fslwiki/flirt/) and the resulting transformations were stored. 8) These transformations were then applied to the streamlines produced in step 6 in order to transform the

streamlines into the MNI152 space. 9) The automated anatomical labelling (AAL) regions of interest (ROIs), [3] which are already in MNI152 standard space, were used to count the number of streamlines between each pair of ROIs. This AAL template consists of 90 ROIs (45 in each hemisphere) covering the entire neocortex (78 cortical ROIs) as well as the subcortical structures amygdala, hippocampus, thalamus, caudate, putamen, and pallidum (12 subcortical ROIs). 10) Streamlines connected to the cerebellum, those running through the brainstem, and streamlines shorter than 5 mm in length were removed (denoted streamlines omitted in Supplementary Table S1). Streamlines that make connections within a ROI itself were deleted (denoted selfloops). In our approach, the strength of a structural connection was operationalized by the number of reconstructed streamlines between two ROIs. 11) The resulting whole-brain connectome was then saved as a fibre density map indicating the number of reconstructed white matter fibres per voxels. These white matter fibre density maps were additionally normalised onto the mean fibre density map of the subjects under investigation using FSL's nonlinear spatial normalisation (FNIRT) tool (http://fsl.fmrib.ox.ac.uk/fsl/fslwiki/fnirt). 12) The fibre density maps were then smoothed with a FWHM Gaussian kernel of 8 mm and subjected to multivariate statistical analyses.

Supplementary Results

Supplementary Table S1: Global brain measures of all Takotsubo syndrome patients and control subjects.

	Takotsubo patients				Control subjects				p-value
**Measures**	**Mean**	**SD**	**Min.**	**Max.**	**Mean**	**SD**	**Min.**	**Max.**	
T1-w. derived global measures (n = 38)									
Intracranial volume (cm^3)	1,455	153	1,233	1,879	1,426	113	1,156	1,729	0.51
Total gray matter volume (cm^3)	530.4	66.6	388.8	678.3	527.4	48.9	432.6	662.0	0.87
Total white matter volume (cm^3)	414.9	67.6	320.1	576.8	409.9	37.4	326.2	507.8	0.78
DTI derived global measures (n = 28)									
Total number of streamlines	2,247,080	292,013	1,774,993	2,885,918	2,163,344	251,567	1,729,653	2,579,869	0.42
Streamlines used to populate matrix	1,005,022	183,845	732,381	1,410,381	970,474	140,658	762,030	1,221,123	0.58
Streamlines omitted	1,298,629	134,857	1,017,459	1,548,227	1,242,898	125,786	998,391	1,412,554	0.27
Selfloops	399,981	88,946	246,268	611,813	363,125	66,324	253,275	469,852	0.23

Measures were compared between groups using t-tests for independent samples. Abbreviations: n, number of subjects; SD, standard deviation.

Supplementary Table S2: Accuracy measures of group classification with a binary support vector machine.

Modality	Measure	Accuracy (%)	p	Sensitivity (%)	p	Specificity (%)	p	ROC AUC	ROC SEM	ROC p	ROC Confidence interval
**DTI**	FA	82.14	0.002	92.86	0.001	71.43	0.065	0.78	0.09	0.012	0.60 – 0.96
(n = 28)	Density	75.00	0.026	78.57	0.023	71.43	0.074	0.83	0.09	0.012	0.60 – 0.96
	AD	64.29	0.087	64.29	0.169	64.29	0.173	0.65	0.11	0.168	0.45 – 0.86
	RD	57.14	0.255	57.14	0.327	57.14	0.337	0.60	0.11	0.383	0.38 – 0.82
	MD	60.71	0.171	64.29	0.016	57.14	0.337	0.61	0.11	0.312	0.40 – 0.83
	all	78.57	0.01	78.57	0.14	78.57	0.26	0.83	0.08	0.003	0.67 – 0.99
**VBM**	GM	47.37	0.600	42.11	0.758	52.63	0.421	0.42	0.10	0.422	0.24 – 0.61
(n = 38)	WM	63.16	0.088	73.68	0.023	52.63	0.432	0.70	0.09	0.032	0.54 – 0.87
	CSF	50.00	0.475	36.84	0.851	63.16	0.148	0.47	0.10	0.759	0.28 – 0.66
	all	57.89	0.15	57.89	0.55	57.89	0.48	0.55	0.10	0.609	0.36 – 0.74
**rsfMRI**	fALFF	75.00	0.009	81.25	0.009	68.75	0.061	0.81	0.09	0.026	0.55 – 0.91
(n = 32)	ALFF	71.88	0.021	75.00	0.017	68.75	0.017	0.82	0.09	0.007	0.61 – 0.95
	ReHo	71.88	0.015	75.00	0.015	68.75	0.046	0.79	0.09	0.024	0.56 – 0.91
	all	78.12	0.01	81.25	0.24	75.00	0.27	0.86	0.07	0.001	0.71 – 1.00
**all modalities** (n = 24)		70.83	0.04	66.67	0.32	75.00	0.36	0.71	0.12	0.083	0.48 – 0.93

P-values are calculated with 1000 permutations for the single modalities, multi-kernel analyses across modalities and measures (all) with 100 permutations each. Abbreviations: AD, axial diffusivity; all, all measures of the respective modality in one model; ALFF, amplitude of low frequency fluctuations; AUC, area under curve; CSF, cerebrospinal fluid; DTI, diffusion tensor imaging; fALFF, fractional ALFF; GM, gray matter; MD, mean diffusivity; n, number of subjects included in the analysis; p, p-value; RD, radial diffusivity; ReHo, regional homogeneity; ROC, Receiver Operating Characteristic; rsfMRI, resting state functional magnetic resonance imaging; VBM, voxel-based morphometry; WM, white matter; Density, WM fibre density.

Supplementary Table S3: Model weights of FA and WM fibre density based group classifications.

	Brain structure	Weight (%)	Size (voxel)	Exp. Ranking
**FA**	Parahippocampal gyrus L	3.48	7,824	1.32
	Paracentral lobule L	3.40	8,764	1.57
	Amygdala L	2.58	1,733	3.39
	Paracentral lobule R	2.50	5,446	3.57
	Hippocampus L	2.19	7,469	5.68
	Supp motor area L	2.14	16,324	5.89
	Supp motor area R	2.14	18,118	5.96
	Precentral gyrus R	2.00	22,127	7.54
	Fusiform gyrus L	1.66	18,010	9.39
	Parahippocampal gyrus R	1.65	8,923	9.21
**WM fibre density**	Paracentral lobule L	9.19	8,764	0.96
	Paracentral lobule R	6.72	5,446	1.93
	Supp motor area R	3.74	18,118	2.89
	Supp motor area L	2.66	16,324	4.18
	Putamen R	2.63	8,510	4.64
	Pallidum R	2.23	2,188	6.21
	Amygdala L	1.99	1,733	8.11
	Putamen L	1.99	7,942	8.18
	Precentral gyrus R	1.98	22,127	7.71
	Hippocampus L	1.88	7,469	8.89

Abbreviations: Exp., expected; FA, fractional anisotropy; L, left; R, right; Supp, supplementary; WM, white matter.

Supplementary Table S4: Model weights of ALFF, fALFF and ReHo based group classifications.

Measure	Brain structure	Weight (%)	Size (voxel)	Exp. Ranking
**ALFF**	Parietal Sup R	2.60	1,541	0.97
	Frontal Mid Orb R	2.18	730	1.97
	Angular L	1.83	1,171	3.59
	Precuneus R	1.80	3,068	4.13
	Lingual R	1.72	2,281	5.78
	Cuneus L	1.72	1,432	5.34
	Paracentral Lobule L	1.64	1,114	8.28
	Parietal Sup L	1.62	1,860	9.03
	Frontal Sup Orb R	1.62	514	9.88
	Insula L	1.61	1,858	10.13
**fALFF**	Parietal Sup R	2.08	1,541	1.03
	Parietal Sup L	1.90	1,860	2.00
	Frontal Mid Orb L	1.74	555	3.31
	Heschl R	1.64	241	4.25
	Parietal Inf L	1.54	2,440	5.25
	Angular L	1.49	1,171	6.25
	Paracentral Lobule R	1.43	720	7.75
	Paracentral Lobule L	1.43	1,114	7.41
	Temporal Pole Mid R	1.39	769	11.50
	Cuneus R	1.37	1,411	11.06
**ReHo**	Parietal Sup R	1.81	1,541	1.53
	Temporal Pole Mid L	1.76	673	2.97
	Frontal Mid Orb R	1.75	730	2.75
	Paracentral Lobule L	1.73	1,114	3.00
	Paracentral Lobule R	1.62	720	5.06
	Precentral L	1.56	3,440	6.25
	Precentral R	1.54	2,735	7.03
	Parietal Sup L	1.51	1,860	8.19
	ParaHippocampal R	1.49	1,127	9.09
	ParaHippocampal L	1.48	961	9.78

Abbreviations: ALFF, amplitude of low frequency fluctuations; Exp., expected; fALFF, fractional ALFF; Inf, inferior; L, left; Mid, middle; Orb, orbital; R, right; ReHo, regional homogeneity; Sup, superior.

Supplementary Table S5: Model weights of VBM based group classification.

Measure	Brain structure	Weight (%)	Size (voxel)	Expected Ranking
**WM volume**	Paracentral lobule L	4.79	9,326	0.97
	Precentral L	2.73	24,605	2.00
	Sup parietal lobule L	2.48	13,778	2.87
	Fusiform gyrus R	2.16	19,088	4.53
	Sup parietal lobule R	2.11	14,025	5.03
	Inf temporal gyrus R	2.01	24,783	6.34
	Supp motor area L	1.93	16,711	7.47
	Inf temporal gyrus L	1.91	20,206	8.26
	Supp motor area R	1.88	18,635	8.42
	Paracentral lobule R	1.80	5,427	10.03

Abbreviations: Inf, inferior; L, left; R, right; Sup, superior; Supp, supplementary; VBM, voxel-based morphometry; WM, white matter.

Supplementary References

1 Yendiki, A., Koldewyn, K., Kakunoori, S., Kanwisher, N. & Fischl, B. Spurious group differences due to head motion in a diffusion MRI study. *Neuroimage* **88**, 79-90, doi:10.1016/j.neuroimage.2013.11.027 (2014).

2 Yendiki, A. *et al.* Automated probabilistic reconstruction of white-matter pathways in health and disease using an atlas of the underlying anatomy. *Front Neuroinform* **5**, 23, doi:10.3389/fninf.2011.00023 (2011).

3 Tzourio-Mazoyer, N. *et al.* Automated Anatomical Labeling of Activations in SPM Using a Macroscopic Anatomical Parcellation of the MNI MRI Single-Subject Brain. *Neuroimage* **15**, 273-289 (2002).
